# Level of physical activity and other maternal characteristics during the third trimester of pregnancy and its association with birthweight at term in South Ethiopia: A prospective cohort study

**DOI:** 10.1371/journal.pone.0236136

**Published:** 2020-07-20

**Authors:** Meseret Legesse, Jemal Haider Ali, Md Dilshad Manzar, Mohammed Salahuddin, Hamid Yimam Hassen

**Affiliations:** 1 School of Public Health, College of Health Sciences, Addis Ababa University, Addis Ababa, Ethiopia; 2 Department of Nursing, College of Applied Medical Sciences, Majmaah University, Al Majmaah, Saudi Arabia; 3 Department of Bio-Molecular Sciences, Pharmacology Division, University of Mississippi, Oxford, Mississippi, United States of America; 4 Department of Primary and Interdisciplinary Care, College of Medicine and Health Sciences, University of Antwerp, Antwerp, Belgium; 5 Global Health Institute, College of Medicine and Health Sciences, University of Antwerp, Antwerp, Belgium; University of Mississippi Medical Center, UNITED STATES

## Abstract

Birthweight continues to be the leading infant health indicator and the main focus of infant health policy. Low birthweight babies are at a higher risk of mortality and morbidity in most low-income countries. However, the physical activity level of pregnant women and its association with low birthweight is not well studied in Ethiopia. To address the above gap, we aimed to examine the maternal physical activity level and other characteristics during the third trimester and its association with birthweight at term in South Ethiopia. A community-based prospective cohort study was conducted among 247 randomly selected women in their third trimester of pregnancy. We measured the physical activity level using the Global Physical Activity Questionnaire, which included the type and level of various categories of activities. Anthropometric measurements of mothers were taken following standard procedures, and birthweight was recorded within 72 hours of delivery. To identify the effect of physical activity level and other maternal characteristics on low birthweight, we performed a multivariable logistic regression analysis. Overall, 111 (47.2%) mothers were engaged in vigorous physical activities during third trimester. The incidence of low birthweight was 21.6% and 9.68% among newborns of mothers who engaged in vigorous and moderate or low physical activity, respectively. The incidence of low birthweight at term was significantly associated with vigorous physical activity [adjusted odds ratio (AOR) = 2.48; 95% confidence interval (CI): 1.01–6.09], prolonged standing [AOR = 3.37; 95% CI: 1.14–9.93], and squatting [AOR = 2.61; 95% CI: 1.04–6.54)] during the third trimester of pregnancy. The vast majority of pregnant women were engaged in vigorous physical activities in their third trimester. Engagement in vigorous physical activity, standing for longer hours, and squatting were the major contributors to low birthweight at term. Hence, focused counseling should be conducted to reduce vigorous physical activity, standing, and squatting during the third trimester among pregnant women.

## Introduction

Low birthweight (LBW) continued to be a significant public health problem, globally, with the highest prevalence in low- and middle-income countries (LMICs). The United Nations Children‘s Fund reported a global LBW rate of 14.6%, and more than 91% of LBW infants are born in LMICs [1]. It is an important public health concern, which influences the child’s health and nutritional status, physical growth, psychosocial development, and survival [2]. It is among the leading predictors of infant mortality within the first month of life [3]. Later in life, LBW is associated with chronic diseases such as high blood pressure, diabetes, coronary heart disease, and renal insufficiency [4–6]. The prevalence of LBW varies across countries and regions, with higher burden in sub-Saharan Africa and Southeast Asia [7, 8]. In Ethiopia, the prevalence of LBW varies across geographical areas, which ranges from 10% to 28% [9–12]. According to the 2016 Ethiopian Demographic and Health Survey (EDHS), 13% of babies in Ethiopia had LBW [13]. In the same breath, a recent meta-analysis in the country also documented a pooled LBW prevalence of 17.3% [14].

The primary causes for LBW are premature birth and intrauterine growth restriction or a combination of both, in addition to maternal attributes, indicating multifactorial etiologies. Socioeconomic factors such as unfavorable socioeconomic conditions, place of residence, age at pregnancy, maternal level of education, and occupation are risk factors for LBW [9, 10, 15–17]. Physical and behavioral characteristics, including low maternal weight at conception, short maternal stature, maternal comorbidities, absence or inadequate prenatal care, unfavorable reproductive history, absence of antenatal care, birth order and interval, multiple pregnancy, and illicit drug use considerably increase the likelihood of LBW [12, 17–20].

Women from poor communities often engage in heavy physical activities at home and in farms, and they usually have unfavorable birth outcomes, such as LBW, among others [21]. According to a Kenyan study, a majority of women spent 78% of their day-time on engaging in physical activities even during pregnancy [22], and presumably, a similar level of activities also prevails in Ethiopia, which has one of the highest prevalence of LBW.

To reduce the prevalence of LBW, Ethiopia has been implementing various intervention strategies under its National Nutrition Program (NNP), and has signed the Scaling Up Nutrition target to reduce undernutrition, focusing on the first 1,000 days [23]. The level of physical activity during pregnancy and its association with birthweight is widely documented in high-income countries; consequently, these countries developed guidelines on the recommended level of physical activity for pregnant women in each trimester of pregnancy. Nonetheless, such studies in Ethiopia are scant; thus, we aimed to examine the type and level of maternal physical activities and other characteristics during the third trimester and their association with birthweight at term to avail evidences for some programmatic initiatives.

## Methods and materials

### Study setting

The study was conducted at the Butajira Health and Demographic Surveillance Site (HDSS) in Butajira woreda, Southern Nations, Nationalities, and Peoples’ Region (SNNPR) of Ethiopia. Butajira woreda is located 130 km away from Addis Ababa, the capital city of Ethiopia. The Butajira HDSS was established in 1986 and covers nine rural and one urban kebeles (lowest administrative unit in Ethiopia) and has an estimated total population of 76,350. This study was conducted from January to June 2017.

### Study design and study participants

We conducted a prospective cohort study among pregnant women in the Butajira HDSS. Pregnant women who were in their third trimester (31 to 34 weeks of gestation) during the study period were enrolled, while those who had diabetes mellitus, hypertension, and previous preterm baby were excluded to avoid confounding effect. After obtaining the data on sociodemographic characteristics, obstetric history, and physical activity level at baseline, mothers who performed vigorous physical activities were considered as the exposed group, and those who performed moderate or low intensity physical activities were considered as the non-exposed group.

### Sample size and sampling procedure

We calculated the sample size using Epi-info version 7 StatCalc sample size calculation package for cohort studies, assuming an estimated LBW prevalence of 9.8% among women in the unexposed group, as reported in SNNPR [13]; a 15% difference between the exposed and unexposed group; an 80% power; and a 95% level of confidence. Adding 10% non-response, the final sample size was determined to be 247 women.

All 10 kebeles in the Butajira HDSS site were selected purposively, considering the better follow-up experience of the population. In order to recruit the participants, we prepared a sampling frame. It constituted a list of names of pregnant women who were thought to be in their third trimester and their detailed information, which included their house number and names of kebeles from the HDSS pregnancy survey list. Using the list, the mothers were visited at home, and based on their last menstrual period (LMP), women in their third trimester, particularly 31 to 34 weeks were identified. The sample size was distributed proportionally based on the number of third trimester mothers living in each kebele. Then, the participants were selected from each cluster using simple random sampling.

### Data collection procedure

Data were collected through face-to-face interview, using a structured and pretested questionnaire, which was adapted from the EDHS and other peer reviewed articles. The height of the mother was measured to the nearest 0.1 cm using a wooden stadiometer with a sliding head bar and the mother on barefoot. Mid-upper arm circumference (MUAC) was also measured to the nearest 0.1 cm on the left arm, using an adult MUAC tape, following standard procedure. Pre-pregnancy weight was self-reported, and current weight was measured using a digital weighing scale. The difference between pre-pregnancy weight and current weight was used for estimating the weight gain during pregnancy. The main outcome, weight of the newborn, was measured by trained data collectors within 72 hours after birth using a digital weighing scale. To assess the level of physical activity, we employed the modified global physical activity questionnaire, standardized for low-income countries [24]. The questionnaire measured the level of physical activity of the women within the last 7 days prior to enrollment and contains a list of activities with different domains, frequencies of each activity per week, and durations per day. The type of physical activity at work, travel, and sports or leisure time activities were assessed. The level was then classified as vigorous activities, moderate activities, walking, and sitting/reclining. Then, we dichotomized women into those who performed vigorous physical activities and those who do not (including moderate and low physical activity level). Positions like standing for longer hours, squatting during routine daily physical activity, and lifting heavy loads were also assessed. We assessed the dietary habit of the mother during pregnancy using a food frequency questionnaire (FFQ). The FFQ was prepared to measure the estimated frequency of consumption for a specific food type within one month prior to the baseline survey.

### Data quality control measures

We conducted a 2-day training on interview techniques, physical activity, and anthropometric measurements. The questionnaire was translated into the local language and reviewed for consistency. We also conducted a pilot test in an adjacent zone called ‘*Silte’*, which has similar socioeconomic, cultural, and geographical characteristics as those of the study area. We performed an internal consistency test using the pilot data, and results showed acceptable level (Cronbach’s alpha = 0.79). The collected data were assessed by field supervisors on a daily basis for incompleteness and/or inconsistencies. The anthropometric and birthweight measurement scales were calibrated frequently.

### Definition of terms

Standing for a long period was defined as standing for more than 3 hours per day on average. Vigorous activities were described as activities that require a large amount of physical effort and largely increase breathing or heart rate (e.g., carrying or lifting heavy loads, digging, and construction work). Meanwhile, moderate activities were described as activities that require moderate physical effort and mildly increase breathing or heart rate (e.g., brisk walking and carrying light loads). Sedentary behavior was defined as sitting or reclining at work or at home; while getting to and from places; travelling in a car, bus, motor, or cart; reading; or watching television.

### Statistical methods

The data, upon checks for completeness and consistency, were entered and cleaned using EpiData version 3.1., and then exported to STATA version 14.0 for further processing and analysis. Of the 247 participants enrolled in this study, 11 (4.5%) did not have information on birthweight and 1 (0.4%) born preterm and thus were removed from the analysis. Descriptive statistics including frequencies with percentage or means and standard deviations (SD) were computed as needed. To determine the wealth index, a principal component analysis of housing infrastructure and ownership of household assets was performed, and the score was consequently divided into five quintiles (lowest, second, middle, fourth, and highest). Depending on their engagement in daily physical activity, mothers were categorized into the vigorous intensity physical activity group and moderate or low intensity physical activity group. A bivariable analysis was carried out to determine the crude association of predictor variables with low birthweight, and based on the results, variables significant at p-value <0.05 were selected for multivariable analysis. To identify the major determinants of LBW, a multivariable logistic regression was performed. The variable antenatal care (ANC) follow up was excluded from the multivariable model due to collinearity with iron folic acid supplementation. The adjusted odds ratio (AOR) with its 95% confidence interval (CI) was used to determine the strength and significance of the association. A p-value of <0.05 was considered statistically significant.

### Participant consent and ethical approval

The protocol of this study was approved by the institutional review board of the College of Health Sciences, Addis Ababa University. Written informed consent and parental assent were obtained from the participants after they received explanations about the possible risks, benefits, issue of confidentiality, voluntarism, and purposes of the study. The study is in compliance with the principles of the declaration of Helsinki.

## Results

A total of 247 women at their third trimester of pregnancy were recruited for this study, and 235 of them were included in the final analysis, yielding a retention rate of 95.1%. Meanwhile, 111 (47.2%) and 124 (52.8%) mothers in the third trimester of pregnancy were included in the vigorous and moderate/low physical activity groups, respectively.

### Socioeconomic characteristics of the mothers

The socioeconomic characteristics of the mothers are presented in Table 1. The mean age of the respondents was 29.1 years (SD: 5.4), ranging from 17 to 45 years. Most (71.1%) of them were rural residents, and 18.6% gave birth to a LBW baby. Over three-quarters (78.3%) of mothers were Muslim, and 152 (64.7%) belonged to the Gurage ethnic group. Nearly half (48.1%) of the mothers and 85 (36.2%) of their husbands had no formal education. Most (81.3%) of them used an improved drinking water source. The incidence of LBW significantly varied across age groups, with a higher incidence (28.0%) observed among the age group of 35 to 45 years (p = 0.02). Similarly, a significant variation was observed in the place of residence (p = 0.03), mothers’ level of education (p = 0.015), husbands’ level of education (p = 0.009), and water source (p = 0.015) between groups.

**Table 1:** Socioeconomic characteristics of pregnant women and the incidence of low birthweight in Butajira, Ethiopia.

Maternal characteristics	Total sample N (%)	Incidence of low birthweight n (%)	p-value ^a^
Total	235 (100)	36 (15.3)	
***Age (y)***			
15–24	45 (19.1)	5 (11.1)	0.02
25–34	140 (59.6)	17 (12.1)	
35–45	50 (21.3)	14 (28.0)	
***Residence***			
Rural	167 (71.1)	31 (18.6)	0.03
Urban	68 (28.9)	5 (7.4)	
***Ethnicity***			
Gurage	152 (64.7)	25 (16.4)	0.69
Silte	52 (22.1)	6 (11.5)	
Others**	31 (13.2)	5 (16.1)	
***Formal education***			
No	113 (48.1)	24 (21.2)	0.015
Yes	122 (51.9)	12 (9.8)	
***Husbands’ formal education***			
No	85 (36.2)	20 (23.5)	0.009
Yes	150 (63.8)	16 (10.7)	
***Water source***			
Improved	191 (81.3)	24 (12.6)	0.015
Non-improved	44 (18.7)	12 (27.3)	
***Wealth quintile***			
Lowest	47 (20.0)	7 (14.9)	0.845
Second	48 (20.4)	9 (18.6)	
Middle	46 (19.6)	8 (17.4)	
Fourth	47 (20.0)	7 (14.9)	
Highest	47 (20.0)	5 (10.6)	

*Other = Catholic, Protestant, and other traditional religions

**Other = Oromo, Amhara, Wolayta, and Hadiya

^a^Pearson’s chi-square test

### Mothers’ obstetric history, behavioral characteristics, and anthropometric measurement

Table 2 displays the obstetric history, behavioral and anthropometric characteristics of the mother and the incidence of low birthweight. Most (84.3%) of the mothers were multigravida, 130 (65.7%) had three or more children, and 23 (9.8%) had a history of miscarriage in their lifetime. More than two-fifths (42.9%) gave birth within an interval of 23 months or less. The vast majorities (90.6%) of mothers attended at least one ANC follow-up, and 176 (74.9%) took iron and folic acid supplementation. Almost all (97.4%) of the mothers consumed coffee, and 79 (33.6%) consumed khat (*Catha Edulis*). Mothers with a history of miscarriage had a significantly higher incidence of LBW (34.8%) than those who did not have a history of miscarriage (13.2%) (p < 0.01). Similarly, the incidence varied depending on the use of ANC (p < 0.05) and iron and folic acid supplementation (p < 0.001). Nearly one-fifth (18.3%) of the mothers had a height of less than 155 cm, and 50 (21.3%) had a MUAC of less than 23 cm. Eleven (4.7%) mothers weighed less than 50 kg, and 25 (10.6%) gained less than 7 kg during pregnancy. The incidence of LBW varied with maternal height (p = 0.04), MUAC (p = 0.02), and pre-pregnancy weight (p = 0.04).

**Table 2:** Obstetric and behavioral characteristics of pregnant women in their third trimester in Butajira Ethiopia.

Obstetric and behavioral characteristics	Total sample N (%)	Incidence of LBW n (%)	p-value
Total	235 (100)	36 (15.3)	
***Gravidity*** ^***a***^			
Multigravida	198 (84.3)	31 (15.7)	0.740
Primigravida	37 (15.7)	5 (13.5)	
***Parity (n = 198)***^***a***^			
1–2 children	68 (34.3)	9 (13.2)	0.498
≥3 children	130 (65.7)	22 (16.9)	
***History of miscarriage*** ^***a***^			
Yes	23 (9.8)	8 (34.8)	0.060
No	212 (90.2)	28 (13.2)	
***Birth interval (months) (n = 196)***^***a***^			
<23	84 (42.9)	15 (17.9)	0.498
≥24	112 (57.1)	16 (14.3)	
***Antenatal care***^***a***^			
Yes	213 (90.6)	29 (13.6)	0.024
No	22 (9.4)	7 (31.8)	
***Iron folic acid supplement*** ^***a***^			
Yes	176 (74.9)	18 (10.2)	<0.001
No	59 (25.1)	18 (30.5)	
***Coffee consumption*** ^***b***^			
Yes	229 (97.4)	35 (15.3)	0.926
No	6 (2.6)	1 (16.7)	
***Khat***^***$***^ ***chewing*** ^***a***^			
Yes	79 (33.6)	13 (16.5)	0.731
No	156 (66.4)	23 (14.7)	
***Maternal height (cm)*** ^***a***^			
<155	43 (18.3)	11 (25.6)	0.039
≥155	192 (81.7)	25 (13.0)	
***Maternal mid-upper arm circumference*** ^***a***^			
<23	50 (21.3)	13 (26.0)	0.018
≥23	185 (78.7)	23 (12.4)	
***Pre-pregnancy weight (n = 61)*** ^***b***^			
<50 kg	11 (4.7)	3 (27.3)	0.035
50–60 kg	41 (17.4)	2 (4.9)	
≥60 kg	9 (3.8)	0 (0.0)	
***Gestational weight gain*** ^***b***^			
≤7 kg	25 (10.6)	3 (12.0)	0.1958
8–12 kg	20 (8.5)	0 (0.0)	
>12 kg	5 (2.1)	1 (25.00)	

^$^ Catha edulis

^a^Pearson’s chi-square test

^b^Fisher’s exact test

### Food types and consumption frequency

The food consumption frequency of mothers during pregnancy is summarized in Table 3. Majority (91.1%) and 29 (12.3%) of the mothers consumed cereal and enset (*Ensete ventricosum*)-based food, respectively, at least once a day. More than half (56.6%) and 24 (10.2%) of them consumed vegetables and fruits at least once a day. A total of 106 (45.1%) and 62 (26.4%) of them consumed meat and milk products, respectively, less than once a month. More than two-fifth (41.7%) and 53 (22.6%) of them consumed egg and legumes, respectively, at least once a day. No significant association was observed in the incidence of LBW and consumption of various foods groups.

**Table 3:** Food consumption frequency among pregnant women in their third trimester in Butajira, Ethiopia.

Food items	Total sample N (%)	Incidence of LBW n (%)	p-value
**Total**	235 (100.0)	36 (15.3)	
***Cereals and bread*** ^***a***^			
≥once/day	214 (91.1)	30 (14.0)	0.077
<once/day	21 (8.9)	6 (28.6)	
***Enset based*** ^***a***^			
≥once/day	29 (12.3)	5 (17.2)	0.8598
4–6 times/week	15 (6.4)	2 (13.3)	
2–3 times in a week	33 (14.0)	5 (15.2)	
Once in a week	76 (32.3)	14 (18.4)	
Twice or less in a month	82 (34.9)	10 (12.2)	
***Vegetables*** ^***a***^			
≥once/day	133 (56.6)	19 (14.3)	0.090
4–6 times/week	43 (18.3)	11 (25.6)	
2–3 times/week	59 (25.1)	6 (10.2)	
***Fruits*** ^***b***^			
≥once/day	24 (10.2)	2 (8.3)	0.4007
4–6 times/week	44 (18.7)	7 (15.9)	
2–3 times/week	38 (16.2)	4 (10.5)	
Once a week	46 (19.6)	11 (23.9)	
Once a month	31 (13.2)	3 (9.7)	
<1 in a month	42 (17.9)	9 (21.4)	
***Meat*** ^***b***^			
≥once/day	13 (5.5)	2 (15.4)	0.2743
4–6 times/week	28 (11.9)	2 (7.1)	
Once a week	33 (14.0)	3 (9.1)	
1–2 times/month	55 (23.4)	7 12.7()	
<1 in a month	106 (45.1)	22 (20.8)	
***Egg*** ^***a***^			
≥once/day	98 (41.7)	15 (15.3)	0.9963
2–3 times/week	137 (58.3)	21 (15.3)	
***Legumes*** ^***b***^			
≥once/day	53 (22.6)	10 (18.9)	0.054
4–6 times/week	4 (1.7)	2 (50.0)	
2–3 times/week	70 (29.8)	5 (7.1)	
Once /week	18 (7.7)	3 (16.7)	
1–2 times/month	20 (8.5)	2 (10.0)	
<1 in a month	51 (21.7)	14 (27.5)	
***Milk*, *cheese*, *and yoghurt*** ^***a***^			
≥once/day	41 (17.4)	2 (4.9)	0.1908
2–3 times/week	75 (31.9)	15 (20.0)	
1–2 times/month	57 (24.3)	9 (15.8)	
<1 in a month	62 (26.4)	10 (16.1)	
***Oils and fat*** ^***a***^			
≤once/day	173 (73.6)	26 (15.0)	0.7634
2–3 times/week	31 (13.2)	4 (12.9)	
Once in a week	31 (13.2)	6 (19.4)	
***Sweets*** ^***a***^			
>once/day	52 (22.1)	8 (15.4)	0.9795
4–6 times/week	67 (28.5)	11 (16.4)	
2–3 times/week	44 (18.7)	7 (15.9)	
Once in a week	72 (30.6)	10 (13.9)	

a = Pearson’s chi-square test

b = Fisher's exact test

### Physical activity level and incidence of low birthweight

A total of 111 (47.2%) mothers were engaged in vigorous physical activities during their third trimester. The mean birthweight for newborns of mothers in the vigorous physical activity group was 2,917 grams (SD: 472), while that of newborns of mothers in the moderate or low physical activity group was 3,024 (SD: 376) grams. The incidence of LBW was significantly higher among mothers in the vigorous activity group 24 (21.6%) than moderate or low activity group 12 (9.7%) (p = 0.011). A total of 63 (26.8%) and 34 (14.5%) mothers had history of squatting and standing for longer hours, respectively, in their third trimester. The incidence of LBW was higher in those who stood for longer time (p = 0.013) and squatted (p = 0.009). The incidence of LBW was 25 (21.2%) and 4 (14.3%) in mothers who walked more than 60 minutes and below 30 minutes per day, respectively (p = 0.217). With regard to the level of sedentary behavior and sleeping, 34 (94.4%) and 26 (72.2%) of the mothers who gave birth to LBW babies were those who sat or reclined for <165 minutes per day (15.7%) and slept ≥8 hours (16.4%), respectively.

### Multivariable analysis of physical activity and other determinants of low birthweight

To identify the independent effects of pattern and intensity of physical activity level on LBW, we employed a multivariable logistic regression model. It showed that the incidence of LBW significantly varied according to the level of vigorous physical activity level, standing for longer hours, walking, and squatting during the third trimester. As expected, water source and iron and folic acid supplementation were also significantly associated with LBW. The probability of LBW was 2.5 times higher for mothers who were involved in vigorous physical activities during the third trimester than those involved in moderate or low physical activities [AOR = 2.48, 95% CI: 1.01–6.09]. Similarly, the likelihood of LBW was 3.4 times higher for mothers who were involved in activities that require prolonged standing than those who were not involved in these types of activities [AOR = 3.37, 95% CI: 1.14–9.93]. The risk of LBW was 2.6 times higher in mothers who performed squatting in their third trimester than those who did not perform squatting [AOR = 2.61, 95% CI: 1.04–6.54]. (Table 4)

**Table 4:** Bivariable and multivariable analysis of the association of physical activity, sociodemographic, and reproductive health characteristics with low birth weight in South Ethiopia.

Variable	Low birthweight		COR [95% CI]	AOR [95% CI]
	Yes n (%)	No n (%)		
***Age group (y)***				
15–24	5 (11.1)	40 (88.9)	0.90 [0.31–2.61]	1.08 [0.33–3.51]
25–34	17 (12.1)	123 (87.9)	1	1
35–45	14 (28.0)	36 (72.0)	2.81 [1.26–9.49]	2.36 [0.82–6.75]
***Residence***				
Rural	31 (18.6)	136 (81.4)	2.87 [1.06–7.73]	2.07 [0.60–7.05]
Urban	5 (7.4)	63 (92.6)	1	1
***Educational status***				
Illiterate	24 (21.2)	89 (78.8)	2.47 [1.17–5.22]	0.92 [0.32–2.61]
Literate	12 (9.8)	110 (90.2)	1	1
***Husband’s educational status***				
Illiterate	20 (23.5)	65 (76.5)	2.58 [1.25–5.30]	1.98 [0.92–4.99]
Literate	16 (10.7)	134 (89.3)	1	1
***Water source***				
Improved	24 (12.6)	167 (87.4)	1	1
Unimproved	12 (27.3)	32 (72.7)	2.60 [1.18–5.74]	3.16 [1.17–8.52]*
***Iron folic acid supplementation***				
Yes	18 (10.2)	158 (89.8)	1	1
No	18(30.5)	41 (69.5)	3.85 [1.84–8.06]	6.13 [2.53–14.84]*
***Mid-upper arm circumference***				
<23	13 (26.0)	37 (74.0)	2.47 [1.15–5.33]	2.01 [0.87–6.21]
≥23	23 (12.4)	162 (87.6)	1	1
***Physical activity level***				
Vigorous activity	24 (21.6)	87 (78.4)	2.57 [1.21–5.43]	2.48 [1.01–6.09]*
Moderate activity	12 (9.7)	112 (90.3)	0.10 [0.05–0.19]	1
***Standing for longer hours***				
Yes	10 (29.4)	24 (70.6)	2.80 [1.20–6.52]	3.37 [1.14–9.93]*
No	26 (12.9)	175 (87.1)	1	1
***Squatting***				
Yes	16 (25.4)	47 (74.6)	2.58 [1.24–5.39]	2.61 [1.04–6.54]*
No	20 (11.6)	152 (88.4)	1	1

*Statistically significant association at p < 0.05

## Discussion

The sustainable development goal targeted to reduce neonatal mortality to at least 12 per 1,000 live births by 2030. Likewise, the Global Nutrition Target also aimed to achieve a 30% reduction in the number of infants born with a weight lower than 2,500 gm by the year 2025. As part of the LBW reduction strategy, it is indispensable to identify the risk factors linked to it in order to develop a targeted intervention. With this background information, we conducted a prospective cohort study among pregnant women engaged in physical activity during their third trimester, to determine the magnitude and its attributors on the incidence of LBW.

Based on our study, the incidence of LBW was 15.32%, which is consistent with the 2016 EDHS national estimate [13]. Moreover, other related studies in the country reported a LBW incidence of 13%–16.5% [10, 25, 26]. Our estimate is lower than that obtained from previous studies performed in countries with a documented prevalence of 22–54% [27–29]. Such discrepancies were expected since most of the above studies were facility based and targeted the high-risk groups. Compared with some previous studies conducted in Gondar and Jimma cities [30, 31], the values observed in the present study were higher due to the variations across geographical settings and seasonality. A recent systematic review performed in Ethiopia documented a pooled estimate of 17.3%, which is slightly higher than our findings [14], suggesting that LBW remains an important public health problem in the country.

In our study, 47.2% of mothers were involved in vigorous physical activities at work or home, and a significant proportion of them took part in activities that required squatting and standing for longer hours during their third trimester. Moreover, being involved in high intensity physical activity along with the warm weather would consequently lead to small for gestational age fetus and low birthweight [32].

In the multivariable analysis, engaging in vigorous intensity physical activities, prolonged standing, squatting during the third trimester, using unimproved water sources, and not taking iron supplements during pregnancy were significant determinants of LBW at term. The incidence of LBW was higher among mothers involved in vigorous activities, and our finding is concordant with McCowan et al.’s findings who documented that daily vigorous physical activity is associated with higher risk of LBW [33]. Bisson et al. also reported a 19.8 gram reduction in birthweight for one metabolic equivalent of task (MET)/hours/week increment spent in any vigorous exercise [32]. Moreover, Magann and his colleagues showed that women who engaged in heavy exercise had smaller (86.5 g) infants than sedentary women, which again emphasizes how vigorous activities affect birthweight [34]. By contrast, few studies indicated no significant effect of vigorous physical activity on birthweight [35–37]. These findings need to be interpreted cautiously since these studies assessed the effects of planned physical activity alone, which was 2 to 3 times per week for not more than 1 hour.

Mothers involved in activities that require prolonged standing were also more likely to have LBW neonates, and this finding is in congruence with the systematic review report, which showed that uninterrupted standing increased the odds of LBW threefold [38]. Increase in the activity of the sympathetic nervous system in active muscles, following prolonged standing results in the return of blood from visceral arteries to active muscles, increased sweating, decreased plasma volume, and reduced perfusion of blood to uterine and placental arteries [39].

The odds of having LBW was higher among mothers who performed squatting and is again in line with the findings of the Thailand and Indian studies, which showed that women who performed activities in squatting position were more likely to give birth to LBW babies [40, 41].

Using water from non-improved source was found to increase the odds of LBW babies, and similar finding was reported by a study from Ghana, which documented that living in a community with low coverage of safe water supply is associated with a high prevalence of LBW [42]. It is likely that mothers who do not have improved water supply at their locality might travel far to bring water. This causes physical strain that could result in low gestational weight gain, a known risk factor for LBW birth.

Mothers who did not receive iron supplements during pregnancy were more likely to have LBW babies. Our result is consistent with some previous studies conducted in Ethiopia [10, 43] and Nepal [44] that showed that by supplementing mothers with iron and other micronutrients, the risk of LBW could be reduced. This is sufficient evidence to support the fact that iron folic acid supplementation is associated with normal birthweight [45].

The following limitations need to be considered when interpreting the findings of this study. First, the use of self-reported physical activity could lead to subjective memory and reporting variations. However, as the variation is non-differential, i.e., it is not dependent on the outcome, the effect size estimate will not be biased. Second, we did not estimate the MET; hence, we could not measure the amount of reduction in birthweight for every unit increase in MET. Third, due to practical difficulty, we did not measure the exact amount of food servings consumed. However, the relative food frequency indicates minimal or no variation in food intake between study groups. Fourth, due to low educational status, only 25% of women reported pre-pregnancy weight. Thus, we could not adjust for pre-pregnancy BMI. Finally, we did not estimate the pregnancy weight gain at each trimester of pregnancy, which might have an impact on birthweight. However, as pregnancy weight gain might be in the causal pathway, controlling for it could lead to a biased estimate. Therefore, we believe that the effect size for vigorous physical activity is still valid.

## Conclusion

The incidence of LBW in Ethiopia is considerably high. A significant proportion of women continue engaging in activities requiring vigorous physical effort, prolonged standing and squatting during the span of their pregnancy. These activities were found to be significantly associated with LBW in their babies. Lack of access to an improved water source and poor iron folic acid supplement utilization were linked with LBW. Therefore, health professionals working in maternity units should perform counseling regarding the recommended level of physical activity in each trimester of pregnancy. Moreover, the National Nutrition Program needs to improve the optimal dose of iron folic acid supplementation for pregnant women. Further research with a larger sample size and objective measurement of physical activity in each trimester is recommended.

## Supporting information

S1 Table STROBE statement—checklist of items for cohort studies. (DOCX)

S1 File The English and Amharic version of the questionnaire used in this study. (PDF)

S1 Dataset The minimal dataset of the study. (ZIP)

## References

[1] United Nations Children’s Fund (UNICEF), World Health Organization (WHO). UNICEF-WHO Low birthweight estimates: Levels and trends 2000–2015. Geneva: World Health Organization, 2019.

[2] Huang C, Martorell R, Ren A, Li Z. Cognition and behavioural development in early childhood: the role of birth weight and postnatal growth. International Journal of Epidemiology. 2012;42(1):160–71. 10.1093/ije/dys207 23243117 PMC3600622

[3] Welaga P, Moyer CA, Aborigo R, Adongo P, Williams J, Hodgson A, et al Why are babies dying in the first month after birth? A 7-year study of neonatal mortality in northern Ghana. PLoS One. 2013;8(3):e58924–e. Epub 2013/03/19. 10.1371/journal.pone.0058924 . 23527050 PMC3602544

[4] Wang T, Huang T, Li Y, Zheng Y, Manson JE, Hu FB, et al Low birthweight and risk of type 2 diabetes: a Mendelian randomisation study. Diabetologia. 2016;59(9):1920–7. Epub 2016/06/24. 10.1007/s00125-016-4019-z 27333884 PMC4970938

[5] Knop Marianne R, Geng TT, Gorny Alexander W, Ding R, Li C, Ley Sylvia H, et al Birth Weight and Risk of Type 2 Diabetes Mellitus, Cardiovascular Disease, and Hypertension in Adults: A Meta‐Analysis of 7 646 267 Participants From 135 Studies. Journal of the American Heart Association. 2018;7(23):e008870 10.1161/JAHA.118.008870 30486715 PMC6405546

[6] Reyes L, Manalich R. Long-term consequences of low birth weight. Kidney International. 2005;68:S107–S11. 10.1111/j.1523-1755.2005.09718.x. 16014086

[7] Blencowe H, Krasevec J, de Onis M, Black RE, An X, Stevens GA, et al National, regional, and worldwide estimates of low birthweight in 2015, with trends from 2000: a systematic analysis. The Lancet Global Health. 2019;7(7):e849–e60. 10.1016/S2214-109X(18)30565-5 31103470 PMC6560046

[8] Mahumud RA, Sultana M, Sarker AR. Distribution and Determinants of Low Birth Weight in Developing Countries. J Prev Med Public Health. 2017;50(1):18–28. Epub 2017/02/09. 10.3961/jpmph.16.087 28173687 PMC5327679

[9] Demelash H, Motbainor A, Nigatu D, Gashaw K, Melese A. Risk factors for low birth weight in Bale zone hospitals, South-East Ethiopia: a case–control study. BMC Pregnancy and Childbirth. 2015;15(1):264 10.1186/s12884-015-0677-y 26463177 PMC4604703

[10] Gebremedhin M, Ambaw F, Admassu E, Berhane H. Maternal associated factors of low birth weight: a hospital based cross-sectional mixed study in Tigray, Northern Ethiopia. BMC Pregnancy and Childbirth. 2015;15(1):222 10.1186/s12884-015-0658-1 26382941 PMC4574616

[11] Talie A, Taddele M, Alemayehu M.—Magnitude of Low Birth Weight and Associated Factors among Newborns Delivered in Dangla Primary Hospital, Amhara Regional State, Northwest Ethiopia, 2017. 10.1155/2019/3587239 PMC6420968 30941217

[12] Wachamo TM, Bililign Yimer N, Bizuneh AD. Risk factors for low birth weight in hospitals of North Wello zone, Ethiopia: A case-control study. PLoS One. 2019;14(3):e0213054 Epub 2019/03/21. 10.1371/journal.pone.0213054 30893344 PMC6426181

[13] Central Statistical Agency (CSA) [Ethiopia], ICF. Ethiopia Demographic and Health Survey 2016. Addis Ababa, Ethiopia, and Rockville, Maryland, USA: CSA and ICF, 2016.

[14] Endalamaw A, Engeda EH, Ekubagewargies DT, Belay GM, Tefera MA. Low birth weight and its associated factors in Ethiopia: a systematic review and meta-analysis. Ital J Pediatr. 2018;44(1):141–. 10.1186/s13052-018-0586-6 . 30477557 PMC6258299

[15] Elster AB. The effect of maternal age, parity, and prenatal care on perinatal outcome in adolescent mothers. Am J Obstet Gynecol. 1984;149(8):845–7. 10.1016/0002-9378(84)90602-1 . 6540522

[16] Khan A, Nasrullah FD, Jaleel R. Frequency and risk factors of low birth weight in term pregnancy. Pak J Med Sci. 2016;32(1):138–42. 10.12669/pjms.321.8120 . 27022362 PMC4795855

[17] Singh G, Chouhan R, Sidhu K. Maternal Factors for Low Birth Weight Babies. Med J Armed Forces India. 2009;65(1):10–2. Epub 2011/07/21. 10.1016/S0377-1237(09)80045-2 . 27408181 PMC4921448

[18] Kramer MS. The Epidemiology of Adverse Pregnancy Outcomes: An Overview. The Journal of Nutrition. 2003;133(5):1592S–6S. 10.1093/jn/133.5.1592S 12730473

[19] Lumbanraja S, Lutan D, Usman I. Maternal Weight Gain and Correlation with Birth Weight Infants. Procedia—Social and Behavioral Sciences. 2013;103:647–56. 10.1016/j.sbspro.2013.10.383.

[20] Oulay L, Laohasiriwong W, Phajan T, Assana S, Suwannaphant K. Effect of antenatal care on low birth weight prevention in Lao PDR: A case control study [version 1; peer review: 1 approved with reservations, 1 not approved]. F1000Research. 2018;7(1138). 10.12688/f1000research.15295.1

[21] Borodulin KM, Evenson KR, Wen F, Herring AH, Benson AM. Physical activity patterns during pregnancy. Med Sci Sports Exerc. 2008;40(11):1901–8. 10.1249/MSS.0b013e31817f1957 . 18845974 PMC3319731

[22] Odiwuor AF, Judith K. Physical Activity Patterns of Pregnant Women in Rongo, Kenya. J Arts Humanit Soc Sci. 2016;4(2):139–46.

[23] FMOH. National Nutrition Programme June 2013-June 2015. Addis Ababa: Government of Federal Democratic Republic of Ethiopia 2013.

[24] Armstrong T, Bull F. Development of the World Health Organization Global Physical Activity Questionnaire (GPAQ). Journal of Public Health. 2006;14(2):66–70. 10.1007/s10389-006-0024-x

[25] Gebremedhin S, Enquselassie F, Umeta M. Independent and joint effects of prenatal Zinc and Vitamin A Deficiencies on birthweight in rural Sidama, Southern Ethiopia: prospective cohort study. PLoS One. 2012;7(12). 10.1371/journal.pone.0050213 PMC3521768 23272058

[26] Teshome D, Telahun T, Solomon D, Abdulhamid I. A study on birth weight in a teaching-referral hospital, Gondar, Ethiopia. The Central African journal of medicine. 2006;52(1–2):8–11. 17892233

[27] Assefa N, Berhane Y, Worku A. Wealth status, mid upper arm circumference (MUAC) and antenatal care (ANC) are determinants for low birth weight in Kersa, Ethiopia. PLoS One. 2012;7(6). 10.1371/journal.pone.0039957 PMC3386987 22792140

[28] Zenebe K, Awoke T, Birhan N. Low birth weight & associated factors among newborns in Gondar town, North West Ethiopia: institutional based cross-sectional study. Indo Global Journal of Pharmaceutical Sciences. 2014;4(2):74–80.

[29] Mekonen HK, Nigatu B, Lamers WH. Birth weight by gestational age and congenital malformations in Northern Ethiopia. BMC pregnancy and childbirth. 2015;15(1):76. 25886401 10.1186/s12884-015-0507-2 PMC4381366

[30] Adane AA, Ayele TA, Ararsa LG, Bitew BD, Zeleke BM. Adverse birth outcomes among deliveries at Gondar University hospital, Northwest Ethiopia. BMC pregnancy and childbirth. 2014;14(1):90. 24576205 10.1186/1471-2393-14-90 PMC3996071

[31] Gebremariam A. Factors predisposing to low birth weight in Jimma Hospital south western Ethiopia. East African Medical Journal. 2005;82(11):554 10.4314/eamj.v82i11.9408 16463748

[32] Bisson M, Croteau J, Guinhouya BC, Bujold E, Audibert F, Fraser WD, et al Physical activity during pregnancy and infant's birth weight: results from the 3D Birth Cohort. BMJ Open Sport Exerc Med. 2017;3(1):e000242–e. 10.1136/bmjsem-2017-000242 . 28761717 PMC5530125

[33] McCowan LME, Roberts CT, Dekker GA, Taylor RS, Chan EHY, Kenny LC, et al Risk factors for small-for-gestational-age infants by customised birthweight centiles: data from an international prospective cohort study. BJOG: An International Journal of Obstetrics & Gynaecology. 2010;117(13):1599–607. 10.1111/j.1471-0528.2010.02737.x 21078055

[34] Magann EF, Evans SF, Weitz B, Newnham J. Antepartum, intrapartum, and neonatal significance of exercise on healthy low-risk pregnant working women. Obstet Gynecol. 2002;99(3):466–72. Epub 2002/02/28. 10.1016/s0029-7844(01)01754-9 . 11864675

[35] Ruchat SM, Davenport MH, Giroux I, Hillier M, Batada A, Sopper MM, et al Walking Program of Low or Vigorous Intensity During Pregnancy Confers an Aerobic Benefit. Int J Sports Med. 2012;33(08):661–6. Epub 17.04.2012. 10.1055/s-0032-1304635 22510805

[36] Jukic AMZ, Evenson KR, Daniels JL, Herring AH, Wilcox AJ, Hartmann KE. A prospective study of the association between vigorous physical activity during pregnancy and length of gestation and birthweight. Matern Child Health J. 2012;16(5):1031–44. 10.1007/s10995-011-0831-8 . 21674218 PMC3386423

[37] Pathirathna ML, Sekijima K, Sadakata M, Fujiwara N, Muramatsu Y, Wimalasiri KMS. Effects of Physical Activity During Pregnancy on Neonatal Birth Weight. Scientific Reports. 2019;9(1):6000 10.1038/s41598-019-42473-7 30979921 PMC6461641

[38] Bisson M, Lavoie-Guénette J, Tremblay A, Marc I. Physical Activity Volumes during Pregnancy: A Systematic Review and Meta-Analysis of Observational Studies Assessing the Association with Infant's Birth Weight. AJP Rep. 2016;6(2):e170–e97. 10.1055/s-0036-1583169 . 27127718 PMC4848034

[39] Ebrahimi M, Bahraninejad Z. The relationship between occupational fatigue and preterm delivery. ISMJ. 2014;17(2):233–42.

[40] Tuntiseranee P, Geater A, Chongsuvivatwong V, Kor-anantakul O. The effect of heavy maternal workload on fetal growth retardation and preterm delivery. A study among southern Thai women. J Occup Environ Med. 1998;40(11):1013–21. Epub 1998/11/27. 10.1097/00043764-199811000-00013 . 9830610

[41] Koushkie Jahromi M, Namavar Jahromi B, Hojjati S. Relationship between Daily Physical Activity During Last Month of Pregnancy and Pregnancy Outcome. Iran Red Crescent Med J. 2011;13(1):15–20. Epub 2011/01/01. . 22946014 PMC3407581

[42] Kayode GA, Amoakoh-Coleman M, Agyepong IA, Ansah E, Grobbee DE, Klipstein-Grobusch K. Contextual risk factors for low birth weight: a multilevel analysis. PLoS One. 2014;9(10):e109333 Epub 2014/11/02. 10.1371/journal.pone.0109333 25360709 PMC4215836

[43] Siyoum M, Melese T. Factors associated with low birth weight among babies born at Hawassa University Comprehensive Specialized Hospital, Hawassa, Ethiopia. Ital J Pediatr. 2019;45(1):48–. 10.1186/s13052-019-0637-7 . 30975170 PMC6460807

[44] Bhaskar RK, Deo KK, Neupane U, Chaudhary Bhaskar S, Yadav BK, Pokharel HP, et al A Case Control Study on Risk Factors Associated with Low Birth Weight Babies in Eastern Nepal. Int J Pediatr. 2015;2015:807373-. Epub 2015/12/10. 10.1155/2015/807373 . 26783406 PMC4689955

[45] Christian P. Micronutrients, Birth Weight, and Survival. Annual Review of Nutrition. 2010;30(1):83–104. 10.1146/annurev.nutr.012809.104813 20415580

