# Supplementary material for: Level of physical activity and other maternal characteristics during the third trimester of pregnancy and its association with birthweight at term in South Ethiopia: A prospective cohort study
Source: PLoS One. 2020 Jul 20;15(7):e0236136. doi: 10.1371/journal.pone.0236136 (PMC7371203; doi:10.1371/journal.pone.0236136)
Supplement: S1 File — (PDF) [file pone.0236136.s002.pdf]

Annex 2: Questionnaire (English Version)

Addis Ababa University School of Public Health

Questionnaire to assess level of daily physical activity of pregnant women during third trimester of pregnancy and its effect on birth weight of a newborn in Butajira, 2017.

Questionnaire ID: ________________________

Address	Kebele ______________House no. _____________
Date of interview	__________/dd/__________/mm/______/yr/____________
Time started	Hour __________minutes _______
Time ended	Hour _________minute ________
Interviewer	Name ______________________________<br>Phone number ________________signature __________
Checked by Supervisor	Name _____________________________________<br>Phone number __________________Signature _______

**Part 1: Socio-demographic and economic Characteristics**			
No.	Question and filters	Response/Choices	Skip
101	How old are you? (Age in complete years)	…………………years	
102	Residence	Urban……………………………1<br>Rural ……………………………2	
103	What is your religion?	Orthodox…………………………..…1<br>Muslim …………………………..…..2<br>Protestant…………………………..……3<br>Catholic …………………………..…4<br>Traditional ……………………….…….5<br>Other (specify)……………………..99	
104	What is your ethnicity?	Gurage ………………………………1<br>Silte …………………………………2<br>Oromo ……………………………..…...3<br>Amhara………………………………4<br>Tigree ……….……………….……......5<br>Wolayita ……………………………6<br>Hadiya ………………………………7	

		Kembata ……………………………..8<br>Others specify ………………………99	
105	What is your current marital status?	Single……….……………………….…1<br>Married ………………….....................2<br>Divorced……………………….........3<br>Separated …………………………..…4<br>Widowed………………….................5<br>Cohabitating ………………………...6	
106	What is your educational status?	Illiterate…………………………….…..1<br>Read and write……………….….…..…2<br>Primary……………………………......3<br>Secondary……………….................4<br>Technical/vocational…………………5<br>Higher (specify)…………………......6	
107	What is your spouse’s educational status?	Illiterate………………………….……..1<br>Read/write…………………….………..2<br>Primary……………….….................3<br>Secondary……………….................4<br>Technical/vocational………………...5<br>Higher (specify)…………………......6	
108	What do you do for a living?(occupational status)<br>(NB. more than one answer is possible)	House wife ……………..……………1<br>Farmer…………………….……………2<br>Merchant…………………………….3<br>Government/private employee……...4<br>Daily laborer……………….............5<br>Student…………………………….....6<br>Other specify…………………….…99	
109	What does your Husband/partner do for a living?(occupational status of your husband/partner)<br><br>(NB. more than one answer is possible)	Farmer…………………………….....1<br>Daily laborer…………………….......2<br>Merchant………………………......3<br>Government/private employee……..4<br>Student…………………………........5<br>other specify ……………….…..…99	
110	What is the main source of drinking water for member of your house holds?	Piped water……………………….....1<br>Protected well…………………..…..2<br>Unprotected well………………........3<br>Protected spring……………….…..4<br>Unprotected spring…………..…..5<br>lake…………………………….…..6<br>river ……………………………......7	

		pond……………….……………..…8<br>other specify……………….………99	
111	Does your household own a toilet facility?	Yes ……………………………..1<br>No ……………………..............2	
112	In total how much is the monthly income of your household?	………………….....Birr	
113	What type of fuel does your household mainly use for cooking?	Electricity……………………..…1<br>Solar……………………………..…2<br>Kerosene…………………………..3<br>Charcoal…………………..…….4<br>Wood………………………..…...5<br>Shrubs/grass……………………..…6<br>Animal dung……………………......7<br>Other specify…………………….....99	
114	What is the main material of the floor of your house?	Mud……………………..………1<br>Sand……………………...……..2<br>Wood……………………………3<br>Cement……………………….…4<br>Marble……………………….….5<br>Cane/bamboo ……………………...6<br>Other (specify)………………..…...99	
115	What is the main material of the roof of your house?	Grass ……………………..……......1<br>Wood…………………….……...2<br>Corrugated iron/metal …………......3<br>Cement……………………………..4<br>Other(specify)……………………......5	
116	What is the main material of the wall of your house?	Wood with mud……………………1<br>Wood with grass…………………...2<br>Blocks…………………………..…3<br>Cement with stone………………....4<br>Bricks…………………………5<br>Corrugated iron/meta …………6<br>Other(specify)…………………7	
117	Does your household have?		

	Electricity?<br>A watch/ clock?<br>A radio?<br>A television?<br>A mobile telephone?<br>Non mobile telephone<br>A refrigerator?<br>A table?<br>A chair?<br>A bed with cotton/sponge/spring mattress?<br>An electric mitad?<br>A kerosene lamp/pressure lamp?<br>A motorcycle?<br>An animal-drawn cart?<br>A boat?<br>A Mill?	Yes No<br>Electricity? 1 2<br>A watch/ clock? 1 2<br>A radio? 1 2<br>A television? 1 2<br>A mobile telephone? 1 2<br>Non mobile telephone 1 2<br>A refrigerator? 1 2<br>A table? 1 2<br>A chair? 1 2<br>A bed with cotton/sponge/spring mattress? 1 2<br>An electric mitad? 1 2<br>A kerosene lamp/pressure lamp? 1 2<br>A motorcycle? 1 2<br>An animal-drawn cart? 1 2<br>A boat? 1 2<br>A Mill? 1 2	
118	Does your house have windows?	Yes ………………..1<br>No ………………..2	
119	Does your household own any land (owned or rented) that can be used for agriculture?	Yes ……………….1<br>No ………………..2	
120	How many of the following animals do this household own?<br>Milk cows or oxen or bulls?<br>Horses, donkeys, or mules?<br>Goats or sheep?<br>Chickens?<br>Beehives?<br>(NB: more than one answer is possible)	Yes No<br>Milk cows or oxen or bulls…… 1 2<br>Horses, donkeys, or mules…… 1 2<br>Goats…………………………. 1 2<br>Sheep ………………………. 1 2<br>Chickens …………………….. 1 2<br>Beehives …………………….. 1 2	

Part 2: Reproductive history

No.	**Questions and filter**	Responses/choices	Skip
201	When did your last menstrual period start?	___/____/____ (dd/mm/yy)	
202	Have you ever given birth before?	Yes …………1<br>No …………..2 →	205

203	How many live births did you give before?<br>*(*Data collector: Register live births only*)*	……………… Child/Children	
204	What is the inter-pregnancy interval of the last pregnancy with the current pregnancy? (Please calculate in months)	Inter-pregnancy interval in months:<br>______________	
205	Have you ever had abortion?	Yes……………..1<br>No………………2 →	301
206	How many times have you ever had abortion?	…………………time/s	
**Part 3: Health Care and Medical Problems During Pregnancy**			
**No.**	**Questions and filters**	**Responses/choices**	**Skip**
301	Have you received ANC services for the current pregnancy?	Yes……………..1<br>No………………2 →	303
302	How many months pregnant were you when you first received ANC for this pregnancy?	________ months<br>I don't remember/Not sure …………9	
303	During this pregnancy did you take Fe-Fol tablets? *(show the picture)*	Yes ……………………1<br>No ……………………..2 →	305
304	How many months pregnant were you when you first take Fe-fol? *(show the picture)*	Yes ……………………………..…1<br>No ………...……………...……..2	
305	During this pregnancy, did you have malaria infection?	Yes …………………………...……1<br>No ………………………..……..2	
306	Do you have any confirmed medical illness?	Yes …………………………….….……1<br>No ……………………………….…....2	
307	During this pregnancy, did you have symptoms of severe headache, generalized body swelling and blurring of vision?	Yes ……………………….………...1<br>No ……………………………….…....2	
**Part 4:Substance use during pregnancy**			
**No.**	**Questions and filters**	**Responses/choices**	**Skip**
401	During this pregnancy did you ever drink coffee?	Yes …………….1<br>No ……………...2 →	403
402	On average, how many cups of coffee do you take in a such typical day?<br>(DC: Estimate in standard 70 ml coffee cup)	……………….. Cups	
403	During this pregnancy did you ever drink alcohol drinks (Beer, Areke, Tela, and Tej)?	Yes…………….1<br>No………………...2 →	405
404	How often were you taking alcohol drinks in the past 30 days?	……………… day/s	

405	During your current pregnancy, did you ever chew Khat?	Yes ………………1<br>No ………………..2 →	407
406	How often were you chewing khat during the past 30 days?	……………. day/s	
407	During your current pregnancy, did you ever smoke cigarettes?	Yes…………………………1<br>No…………………………..2 →	part 5

Part 5:Information Related to Dietary Intake (Food Frequency Questionnaire)

Instruction: (data collector);For each food item listed below, indicate with a checkmark (**√**) the category that best describes the frequency with which the mother usually eats that particular food items in **past one (1) month.**

**501.Food items**	>once/ day	2-3 times/week	4-6 times/week	once/week	twice/ month	Never in a month
Cereals, bread, injera, food made from grains (e.g. made of maize, sorghum, millet, wheat, barley, teff)						
Any potatoes, sweet potatoes, onion, and other foods made from root and tubers?						
Enset and its products (kocho, bulla)						
Any vegetables, dark Green vegetables (kale, lettuce, cabbage)?						
Any fruits (mango, avocado, banana, etc)						
Any beef, lamb, goat, chicken or other organ meat?						
Any eggs						
Any fresh or dried fish, or shell fish?						
Any food made from beans (e.g., kidney beans, haricot beans, field peas, cowpeas, chickpeas others)?						
Any cheese or yogurt, whole milk,						
Any sugar or honey, sweet/soft drinks?						

**Part 6: Modified Global Physical Activity Questionnaire (GPAQ)**			
**Read:** I am going to ask you about the time you spend doing different types of physical activity in the last seven days. Please answer these questions even if you do not consider yourself to be a physically active person. Think first about the time you spend doing work. Think of work as the things that you have to do such as paid or unpaid work, household chores, harvesting food/crops, Terracing, seeking employment. [Insert other examples if needed].In answering the following questions 'vigorous-intensity activities' are activities that require hard physical effort and cause large increases in breathing or heart rate, NB: (interviewer: Please ask the questions carefully and fill up the boxes appropriately).			
Code	Questions	Responses	Skip
601	Does your work involve vigorous-intensity activity that causes large increases in breathing or heart rate?	Yes …...1<br>No……..2	
Please note the number of days a week and time spent each day on the following activities listed below which are considered to be vigorous. Consider only those activities that are carried out by you in the last seven days.			

code	Activities	Number of days/week	Time spent/ day
601a	Carrying, loading or stacking wood	|_____|day	hr. |___|___| min: |___|___|
601b	Drawing water from the well, river and bringing water from other house	|_____|day	hr. |___|___| min: |___|___|
601c	Manual grinding	|_____|day	hr. |___|___| min: |___|___|
601d	Chopping wood-splitting logs	|_____|day	hr. |___|___| min: |___|___|
601e	Plough or digging	|_____|day	hr. |___|___| min: |___|___|
601f	Weeding	|_____|day	hr. |___|___| min: |___|___|
601g	Any other……….(Please specify)	|_____|day	hr. |___|___| min: |___|___|

602	Does your work involve moderate-intensity activity that causes small increases in breathing or heart rate?	Yes ………………1<br>No……………..…..2	skip
Please note the number of days a week and time spent each day on the following activities listed below which are considered to be moderate. Think of only those activities you carried out in the last seven days. 'Moderate-intensity activities' are activities that require moderate physical effort and cause small increases in breathing or heart rate.			
602a	Washing clothes	|_____|days	hr.|___|___|:min |___|___|
602b	Washing dishes	|_____| days	hr.|___|___|:min |___|___|
602c	Sweeping floor (inside or outside house)	|_____| days	hr.|___|___|:min |___|___|
602d	Mopping floor(bend on knees and using hand)	|_____| days	hr.|___|___|:min |___|___|
602e	Drawing and bringing water from outside tap	|_____| days	hr.|___|___|:min |___|___|
602f	Animal care: feeding animals, washing, cleaning barn, etc.)	|_____| days	hr.|___|___|:min |___|___|
602g	Milking cow	|_____|days	hr.|___|___|:min |___|___|
602h	Gardening: watering plants, pruning, sowing seeds, cleaning, etc.	|_____| days	hr.|___|___|:min |___|___|
602i	Patient and elderly care	|_____| days	hr.|___|___|:min |___|___|
602j	Tailoring	|_____| days	hr.|___|___|:min |___|___|
602k	Child care, dressing, bathing, grooming, feeding etc.	|_____| days	hr.|___|___|:min |___|___|
602l	Cooking or food preparation, baking injera, bread,kocho	|_____| days	hr.|___|___|:min |___|___|

602m	Shopping and carrying packages	|_____|days	hr.|___|___|:min |___|___|	
602n	other__________________(Please specify)	|_____| days	hr.|___|___|:min |___|___|	
603	Do you walk to get to and from places?	Yes……………1<br>No ……………2		
List of places		Day/week	Time spent/day	
603a	To work	|_____| days	hr.|___|__|:min |__|___|	
603b	To market	|_____| days	hr.|___|__|:min |__|___|	
603c	To shops	|_____| days	hr.|___|__|:min |__|___|	
603e	To bring children from school	|_____| days	hr.|___|__|:min |__|___|	
603f	To see friends, relatives or others	|_____| days	hr.|___|__|:min |__|___|	
603g	To church, mosque or temple	|_____| days	hr.|___|__|:min |__|___|	
**Recreational Activities**<br>**Read:** The next questions exclude the work and transport activities that you have already mentioned. Now I would like to ask you about sports, fitness and recreational activities (leisure)				
604	Do you do any vigorous-intensity sports, fitness or recreational (leisure) activities that cause large increases in breathing or heart rate like [running] for at least 10 minutes continuously?	Yes……….……….1<br>No ……………..….…2 →		**skip**<br>606
605	In the last seven days, on how many days do you do vigorous-intensity sports, fitness or recreational (leisure) activities?	|_____| days	hr.|___|__|:min |__|___|	
606	Do you do any moderate-intensity sports, fitness or recreational (leisure) activities that causes a small increase in breathing or heart rate such as brisk		Yes ………1<br>No ……….2 →	608

	walking, swimming for at least 10 minutes continuously?		
607	In the last seven days, on how many days do you do moderate-intensity sports, fitness or recreational (leisure) activities	|_____|days	hr.|__|__|:min|__|__|
**Read:** The following question is about sitting or reclining at work, at home, or with friends including time spent (sitting at a desk, sitting with friends, families travelling in car, bus, train, reading, playing cards or watching television) but do not include time spent sleeping.			
608	How much time do you usually spend sitting or reclining on a such typical day?	hr.|____|____|:min |_____|_____|	
609	How many hours do you sleep on average during night?	hr.|____|____|:min |____|_____|	
610	Does your routine activity involve the following?	Yes No	
	Standing for longer hours	1 2	
	Lifting heavy loads	1 2	
	Squatting	1 2	

Thank you for your cooperation

**Part 7. Maternal Anthropometric Measurement**		
701	Height	|__|__|__|. |____|cm
702	MUAC	|__|__|__|. |____|cm
703	Pre pregnancy weight	|___|___|. |____| Kg
704	Gestational weight gain	|___|___|. |____| Kg
**Part 8.Information on newborn (To be filled after delivery)**		
801	Sex of the newborn	Male ………..……..1<br>Female ……………..2

802	Birth weight	|___|___|. |____| grams

Data collector: Name ——————————————————————

Phone number ——————————Signature ————

Checked by Supervisor: Name ——————————————————————

Phone number ——————————Signature ————

Date: dd_______________/mm________yy________________

Annex 4: Questionnaires (Amharic Version)

በአዲስ አበባ ዩኒቨርሲቲ ህክምና ሣይንስ ፋኩልቲ የህብረተሰብ ጤና አጠባበቅ ትምህርት ክፍል እናቶች በእርግዝና ወቅት የሚያደርጉትን የአካል እንቅስቃሴ በሚወልዱት ልጅ ክብደት ላይ ያለውን ተፅዕኖ በተመለከተ የተዘጋጀ የጥናታዊ ፅሁፍ መረጃ መሰብሰቢያ መጠይቅ

የመጠይቁ መለያ ቁጥር ————

አድራሻ	ቀበሌ _______________ የቤት ቁጥር _____________
መጠይቁ የተሞላበት ቀን	_______________/ቀን/___________ /ወር/ _____________ /ዓ.ም/
የተጀመረበት ሰአት	_________________/ሰአት/_________________ /ደቂቃ /
ያለቀበት ሰአት	__________________/ሰአት/ ____________________/ደቂቃ /
ጠያቂ	ስም____________________________ ሰ.ቁ. ____________ ፊርማ _____________
በተቆጣጣሪው ተረጋግጦአል	ስም____________________________ ሰ.ቁ. ____________ ፊርማ _____________

**ክፍል 1፡ተጠያቂዋን የተመለከተ አጠቃላይ መረጃ**			
ተ.ቁ.	ጥያቄዎች	መልስ	እለፍ
101	ዕድሜዎ በሙሉ ዓመት ስንት ነው?	________________________ዓመት	
102	የሚኖሩት የት ነው?	ገጠር ……………………………………1<br>ከተማ………………………..………...2	
103	የምን ሐይማኖት ተከታይ ነዎት?	ኦርቶዶክስ ………………………………1<br>ሙስሊም ………………………………2<br>ፕሮቴስታንት ……………………………..3<br>ካቶሊክ …………………………………4<br>የተለየ ከሆነ ይጠቀስ ……………….……99	
104	የምን ብሔር ተወላጅ ነዎት?	ጉራጌ…………………………………1<br>ስልጤ…………………………………2<br>ኦሮሞ…………………………………3<br>አማራ…………………………………4<br>ትግሬ…………………………………5<br>ወላይታ ………………………………6<br>ሀድያ …………………………………7<br>ከምባታ ………………………………8<br>የተለየ ከሆነ ይጠቀስ ……………………99	
105	የጋብቻ ሁኔታ ?	ያላገቡ…………………………………..1<br>ያገቡ……………………………………2<br>የተፋቱ …………………………………..3<br>የተለያዩ …………………………………4<br>ባል የሞተባቸው …………………………..5	

		ያላገቡ አብረው ሚኖሩ ……………………6	
106	የትምህርት ሁኔታ?	ያልተማሩ ………………………….…1<br>ማንበብና መጻፍ የሚችሉ………….........2<br>አንደኛ ደረጃ …………………………..3<br>ሁለተኛ ደረጃ ……………………………4<br>ቴክኒክ እና ሙያ የተማሩ……………………5<br>ከፍተኛ ትምህርት .. ………………………6	
107	የባለቤትዎ የትምህርት ሁኔታ?	ያልተማሩ ……………………………...1<br>ማንበብና መጻፍ የሚችሉ/……….….……2<br>አንደኛ ደረጃ …………………………...3<br>ሁለተኛ ደረጃ …………………………..4<br>ቴክኒክ እና ሙያ የተማሩ……………………5<br>ከፍተኛ ትምህርት የተማሩ …………………..6	
108	የእርስዎ የስራ ሁኔታ?	የቤት እመቤት ……………………………1<br>አርሶ/አርብቶ አደር ……………….........2<br>ነጋዴ …………………………………...3<br>የመንግሰት/የመ.ያ.ድ. ተቀጣሪ………………..4<br>የጉልበት ሰራተኛ …………………………..5<br>ተማሪ …………………………………...6<br>የተለየ ከሆነ ይጥቀሱ……………….………...99	
109	የባለቤትዎ የስራ ሁኔታ?	አርሶ/አርብቶ አደር ……………….........1<br>ነጋዴ …………………………………...2<br>የመንግሰት/የመ.ያ.ድ.ተቀጣሪ…………….…3<br>የጉልበት ሰራተኛ ………………………….4<br>ተማሪ ……………………………….….5<br>የተለየ ከሆነ ይጥቀሱ………………….…99	
110	በብዛኛውን ጊዜ ቤተሰብዎ የሚጠቀምበት የውሃ መገኛ ምንድነው?	የግል ወይም የጋራ ቧንቧ ……….…………..1<br>የተጠቀ የጉድጓድ ዉሃ ……………………...2<br>ያልተጠበቀ የጉድጓድ ውሃ…………………...3<br>የተጠበቀ የምንጭ ውሃ ……………………..4<br>ያልተጠበቀ የምንጭ ውሃ ……………………5<br>ሐይቅ ……………………………………6<br>ወራጅ ውሃ………………………………..7<br>ኩሬ……………………………………...8<br>ሌላ ከሆነ ይጠቀስ …………………..……99	
111	ቤተሰብዎ የራሱ የሆነ መፀዳጃ ቤት አለው?	አዎን …………………………………1<br>የለም …………………………………2	
112	የቤተሰብዎ ወርሃዊ የገቢ መጠን ምን ያህል ይሆናል?	___________________ ብር	
113	ቤተሰብዎ በአብዛኛው ምግብ ለማዘጋጀት ምን አይነት የሀይል ምንጭ ይጠቀማል?	ኤሌክትሪክ 1	

		የፀሀይ ሀይል ....2<br>ጋዝ/ላምባ..3<br>ከሰል ....4<br>እንጨት...5<br>ጉቶ/ሳር ....6<br>ኩበት /ፍግ ....7<br>የተለየ ከሆነ ይጠቀስ....................................99	
114	ቤተሰብዎ የሚኖርበት ቤት ወለል የተሰራው ከምንድነው?	ከአፈር…………………………………1<br>ከአሸዋ ………………………..………..2<br>ከእንጨት ……………………..………..3<br>ከሲሚንቶ ………………………………4<br>ከእምነበረድ ……………………………5<br>ከሸምበቆ ………………………………..6<br>የተለየ ከሆነ ይጠቀስ……………….……...99	
115	ቤተሰብዎ የሚኖርበት ቤት ጣሪያ የተሰራው ከምንድነው?	ከሳር …………………………………..1<br>ከእንጨት ………………………………2<br>ከቆርቆሮ ………………………………..3<br>ከሲሚንቶ ………………………………4<br>የተለየ ከሆነ ይጠቀስ……………………..…99	
116	ቤተሰብዎ የሚኖርበት ቤት ግድግዳ የተሰራው ከምንድነው?	ከእንጨት እና ከጭቃ …………………….…1<br>ከእንጨት እና ከሳር ……………………….2<br>ከብሎኬት ……………………………….3<br>ከሲሚንቶ እና ከድንጋይ ……………………4<br>ከሸክላ …………………………………..5<br>ከቆርቆሮ …………………………………6<br>የተለየ ከሆነ ይጠቀስ……………...……..99	
117	ከሚከተሉት የትኛው በቤትዎ ይገኛል ወይም አለዎት?		
	ኤሌክትሪክ<br>ሰዓት<br>ሬድዮ<br>ቴሌቪዥን<br>ሞባይል ስልክ<br>ሞባይል ያልሆነ ስልክ<br>ፍሪጅ(ማቀዝቀዣ)<br>ጠረጴዛ<br>ወንበር<br>አልጋ(የጥጥ ወይም የስፖንጅ ፍራሽ ያለው)<br>የኤሌክትሪክ ምጣድ<br>ፋኖስ	አዎን የለም<br>ኤሌክትሪክ 1 2<br>ሰዓት 1 2<br>ሬድዮ 1 2<br>ቴሌቪዥን 1 2<br>ሞባይል ስልክ 1 2<br>ሞባይል ያልሆነ ስልክ 1 2<br>ፍሪጅ(ማቀዝቀዣ) 1 2<br>ጠረጴዛ 1 2<br>ወንበር 1 2<br>አልጋ(የጥጥ ወይም የስፖንጅ ፍራሽ ያለው) 1 2<br>የኤሌክትሪክ ምጣድ 1 2<br>ፋኖስ 1 2	

	ሞተር ሳይክል<br>ጋሪ<br>ጀልባ<br>ወፍጮ	ሞተር ሳይክል 1 2<br>ጋሪ 1 2<br>ጀልባ 1 2<br>ወፍጮ 1 2	
118	ቤትዎ መስኮት አለውን?	አዎን …………………………..1<br>የለም …………………………..2	
119	ቤተሰብዎ ለእርሻ የሚሆን የኪራይ ወይም የይዞታ መሬት አለውን?	አዎን …………………………..1<br>የለም …………………………..2	
120	ቤተሰብዎ ከሚከተሉት ውስጥ የትኛው የቤት እንስሳት አለው?		
	የወተት ላም ወይም በሬ<br>ፈረስ፣አህያ ወይም በቅሎ<br>ፍየል ወይም በግ<br>ዶሮ ወይም ጫጩት<br>ንብ	አዎን የለም<br>የወተት ላም ወይም በሬ 1 2<br>ፈረስ፣አህያ ወይም በቅሎ 1 2<br>ፍየል ወይም በግ 1 2<br>ዶሮ ወይም ጫጩት 1 2<br>ንብ 1 2<br>የተለየ ከሆነ ይጠቀስ ______________________	
**ክፍል 2፡ ስነ-ተዋልዶን የተመለከቱ ጥያቄዎች**			
ተ.ቁ.	ጥያቄዎች	መልስ	እለፍ
201	የመጨረሻውን ያልተዛባ የወር አበባ ያዩበት ቀን መቼ ነበር?	________/________/_________/ቀቀ/ወወ/ዓ.ም	
202	ከዚህ በፊት ልጅ ወልደው ያውቃሉ?	አዎን …………………………..1<br>የለም …………………………..2 →	205
203	እስካሁን በጠቅላላው ስንት ልጅ ወልደዋል?<br>(መረጃ ሰብሳቢ፡በሕይወት የተወለዱትን ብቻ ይመዝግቡ)	_________________________ልጅ/ልጆች	
204	በአሁኑ እና በመጨረሻው እርግዝና መካከል ያለው ርቀት ምን ያህል ነው?(መረጃ ሰብሳቢ፡በወራት ያስሉት)	______________________ወር	
205	ውርጃ አጋጥሞዎት ያውቃል?	አዎን ……………………..1<br>የለም ……………….……..2 →	301
206	ውርጃ ስንት ጊዜ አጋጥሞዎት ያውቃል ?	___________________ጊዜ	
**ክፍል 3፡ ህመምና የጤና አገልግሎትን የተመለከቱ ጥያቄዎች**			
ተ.ቁ.	ጥያቄዎች	መልስ	እለፍ
301	በዚህ እርግዝና የእርግዝና ክትትል አድርገዋል?	አዎን ……….…………………..1<br>የለም ………………………….. →	303
302	በዚህ እርግዝና ለጀመጀመሪያ ጊዜ የእርግዝና ክትትል ሲያደርጉ የስንት ወር እርጉዝ ነበሩ?	_______________ወራት<br>አላስታውስም/እርግጠኛ አይደለሁም …….9	
303	በዚህ እርግዝና የአይረን/ፎሌት እንክብል ወስደዋልን?	አዎን ……….…………………..1<br>የለም …………………………..2 →	305
304	በዚህ እርግዝና የአይረን/ፎሌት እንክብል መውሰድ ሲጀምሩ	__________________ ወር	

	የስንት ወር ነፍሰጡር ነበሩ?	አላስታውስም/እርግጠኛ አይደለሁም ……9	
305	በዚህ እርግዝና በወባ ህመም ተጠቅተው ነበርን?	አዎን …………………………..1<br>የለም …………………………..2	
306	በሀኪም የተረጋገጠ ማንኛውም ህመም አለብዎትን?	አዎን …………………………..1<br>የለም …………………………..2	
307	በዚህ እርግዝና ከባድ የራስ ምታት፣አጠቃላይ የሰውነት ማበጥ እና አይን ላይ ብዥ ማለት አጋጥሞዎት ያውቃል?	አዎን …………………………..1<br>የለም …………………………..2	
**ክፍል 4፡የአኗኗርና ግላዊ ልምዶችን የተመለከቱ ጥያቄዎች**			
ተ.ቁ	ጥያቄዎች	መልስ	እለፍ
401	በዚህ እርግዝና ቡና ጠጥተው ያውቃሉ?	አዎን …………………………..1<br>የለም …………………………..2 →	 403
402	በቀን በአማካይ ስንት ሲኒ ቡና ይጠጣሉ?<br>(**መረጃ ሰብሳቢ**፡መጠኑን በ70 ሚሊ. በመካከለኛ የቡና ሲኒ ይተምኑ)	____________________ሲኒ	
403	በዚህ እርግዝና አልኮልነት ያላቸው መጠጦች(ቢራ፣ጠላ፣አረቄ፣ ጠጅ፣ወይን) ጠጥተው ያውቃሉ?	አዎን …………………………..1<br>የለም …………………………..2 →	 405
404	ባለፉት 30 ቀናት ምን ያህል ጊዜ የአልኮል መጠጦችን ጠጥተው ያውቃሉ?	____________________ጊዜ	
405	በዚህ እርግዝና ጫት ቅመው ያውቃሉ?	አዎን ………………………..1<br>የለም ………………………...2 →	407
406	ባለፉት 30 ቀናት ምን ያህል ጊዜ ጫት ቅመው ያውቃሉ?	________________ቀን	
407	በዚህ እርግዝና ሲጋራ አጭሰው ያውቃሉ?	አዎን …………………………..1<br>የለም …………………………..2 →	 ክፍል 5
**ክፍል 5፡የምግብ አመጋገብን የተመለከቱ ጥያቄዎች**			

መግለጫ፡አሁን ከተዘረዘሩት የምግብ አይነቶች ባለፈው አንድ ወር ውስጥ ስንት ጊዜ ተመግበው እንደነበር እንጠይቆታለሁ፡፡ (መረጃ ሰብሳቢ በምላሻቸው መሰረት (√) ምልክት ያስቀምጡ)

501 የምግብ አይነት ዝርዝር	በቀን ከ1 ጊዜ በላይ	በሳምንት 1ወይም2 ጊዜ	በሳምንት 3-6 ጊዜ	በወር 2 ጊዜ	በወር ከ 2 ጊዜ ያነሰ	በፍፁም አልበላሁም
ዳቦ፣ እንጀራ፣ወይም ከጥራጥሬ ወይም እንደ በቆሎ፣ማሽላ፣ ዘንጋዳ፣ዳጉሳ፣ ስንዴ፣ገብስ፣ጤፍ የተዘጋጀ ምግብ						
ከድንች፣ ስኳር ድንች፣ሽንኩርት እና ሌሎች ስራስሮች የተዘጋጀ ምግብ						
እንስትን (ቆጮ፣ቡላ) የተዘጋጀ ምግብ						
ከአትክልት፣ አረንጓዴ ቅጠላቅጠል፣ሰላጣ፣ጥቅል ጎመን፣የሀበሻ ጎመን የተዘጋጀ ምግብ						

ማንኛውም ፍራፍሬ (ማንጎ፣አቮካዶ፣ፓፓያ፣ሙዝ)						
ማንውም ከበሬ ስጋ፣ከበግ ስጋ፣ከፍየል ስጋ፣ከዶሮ ስጋ የተዘጋጀ ምግብ						
ማንኛውም ከእንቁላል የተዘጋጀ ምግብ						
ማንኛውም ጥሬ ወይም የተጠበሰ ዓሳ						
ከባቄላ፣ ሽንብራ፣አተር፣ በሎቄ፣ጓያ የተዘጋጀ ምግብ						
አይብ፣እርጎ፣ወተት						
ዘይት፣ቅቤ፣ስብ ያለበት ምግብ						
ማንኛውንም ስኳር፣ ማር፣ጣፋጭ፣ለስላሳ መጠጦች						

ክፍል 6፡ የአካል እንቅስቃሴዎችን የተመለከቱ መረጃዎች

የሚነበብ፡በመቀጠል ባለፈው አንድ ሳምንት ውስጥ ስላደረጉአቸው የተለያዩ አካላዊ እንቅስቃሴዎች እጠይቆታለሁ፡፡ እባክዎን ራስዎን አካላዊ እንቅስቃሴ የሚያደርግ ሰው አድርገው ባይቆጥሩም ሁሉንም ተግባራት በመመልከት ጥያቄዎቹን ይመልሱ፡፡ እነዚህም በቤት ውስጥ በመስሪያ ቤት ወይም ከቦታ ወደ ቦታ ለመሄድ የሚያደርጉአቸውን መደበኛ እንቅስቃሴዎች እና በዕረፍት ጊዜዎ ውስጥ ለመዝናኛ ወይም ለስፖርት የሚሰሯቸውን እንቅስቃሴዎች ያጠቃልላሉ፡፡

ጥያቄዎቹን በሚመልሱበት ወቅት ጠንካራ የአካላዊ እንቅስቃሴዎች ማለት ከባድ ጥረት የሚጠይቁ ትንፋሽዎና የልብ ምትዎ ላይ ከፍተኛ ጭማሪ የሚያመጡ ማለትም ቶሎ ቶሎ መተንፈስ ወይም ፈጣን የልብ ምት ሊያስከትሉ የሚችሉ እንቅስቃሴዎች ናቸው፡፡ መካከለኛ የአካላዊ እንቅስቃሴዎች ደግሞ መካከለኛ ጥረት የሚጠይቁ ትንፋሽና የልብ ምት ላይ መጠነኛ ጭማሪ ሊያመጡ የሚችሉ አካላዊ እንቅስቃሴዎች ናቸው፡፡

መለያ	**ጥያቄዎች**	**መልስ**	**እለፍ**
601	መደበኛ አካላዊ እንቅስቃሴዎ ወይም የቤት ውስጥ ስራዎ ከፍተኛ የትንፋሽ ወይም የልብ ምት መጨመር የሚያመጡ ጠንካራ አካላዊ ተግባራትን ያካተተ ነበር?	አዎን ……………………1<br>የለም …………………...2	

ከዚህ በታች የተዘረዘሩት ተግባራት ጠንካራ አካላዊ እንቅስቃሴዎች እንደሆኑ ይታመናል፡፡ እባክዎ እርስዎ እነዚህን እንቅስቃሴዎች በሳምንት ውስጥ ለምን ያህል ቀን እንዳከናወኗቸው እና በአንዱ ቀን ለስንት ሰዓት ያህል እንዳከናወኗቸው ያስቡ፡፡ ሁሉም እንቅስቃሴዎች እርስዎ የሰሯቸው ብቻ መሆን አለባቸው፡፡

<table>
<tr><td colspan="2">የተግባራት ዝርዝር</td><td>በሳምንት ውስጥ የሰሩበት የቀናት ብዛት</td><td>በቀን ውስጥ የወሰደው ጊዜ</td><td>እለፍ</td></tr>
<tr><td>601ሀ</td><td>እንጨት፣ ኩበት መልቀም፣ሸክም መሸከም?</td><td>|_____| ቀናት</td><td>ሰአት : |__|__|ደቂቃ:|__|__|</td><td></td></tr>
<tr><td>601ለ</td><td>ከጉድጓድ ወይም ከወንዝ ውሃ መቅዳት ከአንድ ቤት ወደ ሌላ ቤት ማመላለስ</td><td>|_____| ቀናት</td><td>ሰአት :|__|__|ደቂቃ:|__|___|</td><td></td></tr>
<tr><td>601ሐ</td><td>ጥራጥሬ መፍጨት(ብቅል፣አሻሮ መፍጨት፣አተር፣ባቄላ መከካት)</td><td>|_____| ቀናት</td><td>ሰአት : |__|__|ደቂቃ :|__|__|</td><td></td></tr>
<tr><td>601መ</td><td>እንጨት መፍለጥ፣መሰነጣጠቅ</td><td>|_____| ቀናት</td><td>ሰአት : |__|__|ደቂቃ :|__|__|</td><td></td></tr>
<tr><td>601ሠ</td><td>እህል መውቀጥ</td><td>|_____| ቀናት</td><td>ሰአት : |__|__|ደቂቃ :|__|__|</td><td></td></tr>
<tr><td>601ረ</td><td>ማረስ ወይም መቆፈር፣ መኅልኅል፣መንቀል</td><td>|_____| ቀናት</td><td>ሰአት : |__|__|ደቂቃ :|__|__|</td><td></td></tr>
<tr><td>601ሰ</td><td>ማረም</td><td>|_____| ቀናት</td><td>ሰአት : |__|__|ደቂቃ :|__|__|</td><td></td></tr>
</table>

601ሸ	ሌላ ካለ ይጥቀሱ_______________________________	|_____| ቀናት	ሰአት : |__|__|ደቂቃ :|__|__|	
602	በቤት ወይም ከቤት ውጪ የሚያደርጉት መደበኛ እንቅስቃሴ ወይም የቤት ውስጥ ስራዎች መጠነኛ የትንፋሽና የልብ ምት መጨመር የሚያስከትሉ መካከለኛ አካላዊ ተግባራትን ይጨምራል?	አዎን …………1<br>የለም ……..…..2		
ከዚህ በታች የተዘረዘሩት ተግባራት መጠነኛ የትንፋሽና የልብ ምት መጨመር የሚያስከትሉ መካከለኛ አካላዊ እንቅስቃሴዎች እንደሆኑ ይታመናል፡፡ እባክዎን እርስዎ እነዚህን እንቅስቃሴዎች በሳምንት ውስጥ ለምን ያህል ቀን እንዳከናወኗቸው እና በአንዱ ቀን ለስንት ሰዓት ያህል እንዳከናወኗቸው ያስቡ፡፡ ሁሉም እንቅስቃሴዎች እርስዎ የሰሯቸው ብቻ መሆን አለባቸው፡፡				
መለያ	የተግባራት ዝርዝር	በሳምንት ውስጥ የሰሩበት የቀናት ብዛት	የወሰደው ጊዜ	እለፍ
602ሀ	ልብስ ማጠብ	|_____| ቀናት	ሰአት : |__|__|ደቂቃ :|__|__|	
602ለ	እቃ ማጠብ	|_____| ቀናት	ሰአት : |__|__|ደቂቃ :|__|__|	
602ሐ	ቤት እና ግቢ መጥረግ	|_____| ቀናት	ሰአት : |__|__|ደቂቃ :|__|__|	
602መ	ቤት መወልወል (ቆመው፣አጎንብሰው ወይም ተንበርክከው)	|_____| ቀናት	ሰአት : |__|__|ደቂቃ :|__|__|	
602ሠ	ከቤት ውጪ ከሚገኝ ቧንቧ ውሃ መቅዳት እና ወደ ቤት ማስገባት	|_____| ቀናት	ሰአት : |__|__|ደቂቃ :|__|__|	
602ረ	እንስሳት መንከባከብ (እንስሳቱን ማጠብ፣መመገብ፣በረት ወይም ጋጣ ማፅዳት	|_____| ቀናት	ሰአት : |__|__|ደቂቃ :|__|__|	
602ሰ	ላም ማለብ	|_____| ቀናት	ሰአት : |__|__|ደቂቃ :|__|__|	
602ሸ	አትክልት መንከባከብ(ውሃ ማጠጣት፣በከርከም፣ዘር መዝራት፣ ማፅዳት)	|_____| ቀናት	ሰአት : |__|__|ደቂቃ :|__|__|	
602ቀ	የታመመ ወይም አዛውንት ሰው መንከባከብ	|_____| ቀናት	ሰአት : |__|__|ደቂቃ :|__|__|	
602በ	ልብስ መስፋት (ጥልፍ፣እጅ ስራ መስራት)	|_____| ቀናት	ሰአት : |__|__|ደቂቃ :|__|__|	
602ተ	ልጆች መንከባከብ ልብስ ማልበስ፣ማጠብ፣መመገብ፣አልፎ አልፎ ማቀፍ ወይም መሸከም	|_____| ቀናት	ሰአት : |__|__|ደቂቃ :|__|__|	
602ቸ	ምግብ ማብሰል(ወጥ መስራት፣ቆጮ፣ እንጀራ ፣ዳቦ መጋገር)	|_____| ቀናት	ሰአት : |__|__|ደቂቃ :|__|__|	
602አ	ሱቅ፣ሱፐርማርኬት ወይም ገበያ ውስጥ ተዘዋውሮ መገበየት	|_____| ቀናት	ሰአት : |__|__|ደቂቃ :|__|__|	
602ነ	ሌላ ካለይዘርዝሩ_____________________________	|_____| ቀናት	ሰአት : |__|__|ደቂቃ :|__|__|	
ቀጥሎ ያሉት ጥያቄዎች ከላይ የጠቀሷቸውን በቤት ወይም በህዝብ የስፖርት ስፍራዎች ያደረጉትን መደበኛ እንቅስቃሴዎች አያካትቱም፡፡ በተለምዶ ከቦታ ቦታ የሚጓጓዙባቸውን ለምሳሌ ከቤት ወደ ገበያ ቦታ፣ ወደ አምልኮ የመሳሰሉ ቦታዎች በእግር የሚያደርጉትን ጉዞ የተመለከቱ ናቸው፡፡				
መለያ	ጥያቄ	መልስ		እለፍ
603	ከአንድ ቦታ ወደ ሌላ ቦታ ለመጓዝ በእግር ጉዞ ያደርጋሉ?	አዎን ………………………1<br>አላደርግም ………………….2 →		604
መለያ	የሚሄዱባቸው ቦታዎች ዝርዝር	በሳምንት ውስጥ የሚጓዙበት የቀናት ብዛት	የወሰደው ጊዜ	እለፍ
603ሀ	ወደ ስራ	|_____| ቀናት	ሰአት : |__|__|ደቂቃ :|__|__|	
603ለ	ወደ ገበያ	|_____| ቀናት	ሰአት : |__|__|ደቂቃ :|__|__|	
603ሐ	ወደ ሱቅ	|_____| ቀናት	ሰአት : |__|__|ደቂቃ :|__|__|	
603መ	ልጆች ከትምህርት ቤት ለማምጣት	|_____| ቀናት	ሰአት : |__|__|ደቂቃ :|__|__|	
603ሠ	ዘመድ፣ጓደኛ ለመጠየቅ	|_____| ቀናት	ሰአት : |__|__|ደቂቃ :|__|__|	

603ረ	ወደ ቤተክርስቲያን፣መስኪድ	|____| ቀናት	ሰአት :|__|__|ደቂቃ :|__|__|	
603ሰ	ሌላ ካለ ይዘርዝሩ……………………………………	|____| ቀናት	ሰአት : |__|__|ደቂቃ :|__|__|	

ቀጥሎ ያሉት ጥያቄዎች ከላይ የጠቀሷቸውን በቤት/ በስራ ቦታ ወይም በህዝብ የስፖርት ስፍራዎች ያደረጉትን መደበኛ እንቅስቃሴዎች እና የመጓጓዣ ሁኔታ አያካትትም፡፡ አሁን የምንጠይቆት የመዝናኛ ተግባራትን ለምሳሌ በትርፍ ጊዜዎ በመስሪያ ቤት የእረፍት ሰአት ወይም በቤት ውስጥ ስለሚያደርጓቸው የመዝናኛ እንቅስቃሴዎች ነው፡፡

መለያ	ጥያቄ	መልስ	እለፍ
604	እንደ መዝናኛ/ የትርፍ ጊዜ እንቅስቃሴዎች ከፍተኛ የትንፋሽ ወይም የልብ ምት መጨመር የሚያመጡ ጠንካራ አካላዊ እንቅስቃሴዎችን ቢያንስ ለተከታታይ 10 ደቂቃ ያደርጋሉ? ለምሳሌ፡ ዳንስ (ጭፈራ)፣ ሰምሰማ ሩጫ	አዎን …………1<br>አላደርግም ……..2	→ 606
605	በሳምንቱ ለምን ያህል ቀናት ጠንካራ አካላዊ እንቅስቃሴዎችን ወይም የመዝናኛ/ የትርፍ ጊዜ እንቅስቃሴዎችን አድርገዋል?	|_____| ቀናት |___|___|ደቂቃ :|____|____| ሰአት	
606	እንደ መዝናኛ እንቅስቃሴ መጠነኛ የትንፋሽ ወይም የልብ ምት መጨመር የሚያመጡ መካከለኛ አካላዊ እንቅስቃሴዎችን ቢያንስ ለተከታታይ 10 ደቂቃ አድርገዋል? ለምሳሌ፡ ዋና	አዎን …………1<br>አላደርግም ……..2	→ 606
607	በሳምንቱ ለምን ያህል ቀናት መካከለኛ አካላዊ እንቅስቃሴዎችን ወይም የመዝናኛ/የትርፍ ጊዜ እንቅስቃሴዎችን አድርገዋል?	|_____| ቀናት |_____|____|ደቂቃ :|_____|____| ሰአት	

የሚቀጥለው ጥያቄ በቤት፣በመስሪያ ቤት ወይም በትርፍ ጊዜዎ በመቀመጥ ወይም ጋደም ብለው ያሳለፉትን ጊዜ ይመለከታል፡፡ (ቤት ውስጥ፣ በመኪና ውስጥ ፣ከቤተሰብዎ፣ ከዘመድዎ፣ከጓደኞችዎ ጋር ቴሌቭዥን ለመመልከት ተቀምጠው ወይም ጋደም ብለው ያሳለፉትን ጊዜ ይጨምራል፡፡ ነገር ግን በእንቅልፍ ያሳለፉትን ጊዜ አያካትትም፡፡

608	ከሳምንቱ አንዱን ቀን በጠቅላላው ምን ያህል ጊዜ ተቀምጠው ወይም ጋደም ብለው አሳልፈዋል?	|____|___|ደቂቃ :|____|____| ሰአት	
609	በአንድ ቀን በአማካይ ለስንት ሰዓት ይተኛሉ ?	|____|___|ደቂቃ :|____|____| ሰአት	
610	ከሚከተሉት መካከል በየዕለት ከዕለት እንቅስቃሴዎ ውስጥ የትኞቹን ያደርጋሉ?	አደርጋለሁ	አላደርግም
	ለረጅም ሰአት መቆም	1	2
	ከባድ ዕቃ ማንሳት	1	2
	መንበርከክ /ቁጢጥ ብሎ መስራት/	1	2

ስለ ትብብርዎ እናመሰግናለን

**ክፍል 7. የእናት የሰውነት ልኬት የተመለከቱ መረጃዎች**		
701	ቁመት	|___|___|.|___| ሴ.ሜ.
702	የላይኛው ክንድ ዙሪያ	|___|___|.|___|ሴ.ሜ.
703	ከማርገዝዎ በፊት የነበረዎት ክብደት መጠን	|___|___|.|___| ኪ.ግ
704	በእርግዝና ወቅት የጨመሩት ክብደት መጠን	|___|___|. |___| ኪ.ግ
**ክፍል 8.የተወለደውን/ችውን ህፃን የተመለከቱ መረጃዎች (ከወሊድ በኋላ የሚሞላ)**		
801	ጾታ	ወንድ…..……..……1<br>ሴት ………..……..2
802	ክብደት	|___|___|. |____| ግራም

መረጃ ሰብሳቢ	ስም__________________________ ስ.ቁ. ____________ ፊርማ ____________
በተቆጣጣሪው ተረጋግጦአል	ስም__________________________ ስ.ቁ. ____________ ፊርማ ____________<br>ቀን ___________/ወር___________ /ዓ.ም___________
