# Supplementary material for: Level of physical activity and other maternal characteristics during the third trimester of pregnancy and its association with birthweight at term in South Ethiopia: A prospective cohort study
Source: PLoS One. 2020 Jul 20;15(7):e0236136. doi: 10.1371/journal.pone.0236136 (PMC7371203; doi:10.1371/journal.pone.0236136)
Supplement: Attachment — Submitted filename: Comments to the authors PONE-D-20-09704 May2020.doc [file pone.0236136.s004.doc]

**Level of physical activity during the third trimester of pregnancy and its association with birthweight at term in South Ethiopia: A prospective cohort study**

**PLOS ONE**

**Comments to the authors**

The manuscript reports a prospective cohort study addressing the association of the level of physical activity with birthweigh in Ethiopia, where there is a high incidence of low birthweigh, and women work and house conditions usually lead to high volumes and intensities of physical activity. The study is well designed, the methodology is well explained, and the paper is well written and with a good level of English. Every section contains the necessary information and they are well related to each other. Just a few comments and small corrections have been suggested:

**OVERVIEW:**

The study seems quite complete. However, even if physical activity is the main outcome, I believe the study covers much more than physical activity. The study provides additional information about how other factors may be associated with low birthweigh, such as socioeconomic, obstetric and behavioral characteristics, food consumption and iron and folic acid supplementation. Therefore, I would extend the title of the paper to show that other factors have been studied, for instance: “Level of physical activity and other maternal characteristics during the third trimester of pregnancy and its association with birthweight at term in South Ethiopia: A prospective cohort study”. If the suggestion is followed, I would also mention this in the abstract.

**METHODS**

- Please, explain when this study was carried out.
- Please, explain if the modified version of the global physical activity questionnaire was validated or published somewhere. Was the pilot test in Silte published?
- Does the questionnaire consider the intensity of the physical activity developed? Or was the intensity deduced according to the type of exercise? “The level of physical activity was classified as moderate activity, vigorous activity, leisure time activities, sitting/reclining, walking, and sleeping”. How were low intensity activities classified? Was taking for granted that all leisure time activities were low intensity activities?
- “We assessed the dietary habit of the mother using a food frequency questionnaire within a month, prior to the baseline survey”. Does the food questionnaire used reflect the food habits of the study population? Please, briefly clarify it, as it seems you did.
- “Depending on their engagement in daily physical activity, mothers were categorized into the moderate intensity physical activity group and vigorous intensity physical activity group”. Were low activity women classified as moderate? Or there were no women who developed a low quantity of physical activity? Please, clarify.
- Is it possible to include information about how much activity or intensity was considered to classify women as moderate or vigorous in the methods section?

**RESULTS**

- Please, the first time ANC is mentioned (Mothers’ obstetric history, behavioral characteristics, and anthropometric measurement section), clarify what this means (antenatal consultation?).
- I strongly suggest adding Body Mass Index (BMI) in the anthropometric measurement. Given that you have the height and the pre-pregnancy weigh, it is easy to calculate it, and it could add additional information that may be more representative of the reality than only the height or the weigh by itself.
- I also suggest adding information about BMI in Table 2 to see whether there were more LBW babies among women in any of the BMI categories (maybe underweight?). Even if the aim of the study is to analyze the possible correlation of the level of physical activity with birtweight, the study seems quite complete and provides extra information about socioeconomic, obstetric and behavioral characteristics, and food consumption; therefore BMI could add additional information, since it usually have a strong correlation with physical exercise and nutrition.
- Please, change name of Table 2 (it has been called Table 1 by mistake).
- What was the reason why there is so little information about gestational weight gain? It seem one of the reasons is because little women reported their pre-gestational weight. Please, explain it.
- Table 3: please, modify the order in the enset, vegetables consumption, fruits, legums and sweets rows (4-6 times/week before 2-3 times/week).
- Please, revise and correct the third sentence of the section called “Physical activity level and incidence of low birthweight”, It is mentioned that “The incidence of LBW was significantly higher among mothers in the vigorous activity group 12 (21.6%) than moderate 12 (9.7%) activity group (p = 0.011)”. I believe you mean 24 women in the vigorous activity group; otherwise the numbers do not fit.
- Please, add the statistical significance of the next sentence: “The incidence of LBW was 25 (21.2%) and 4 (11.1%) in mothers who walked more than 60 minutes and below 30 minutes per day, respectively (, ADD with no statistical significance?)”.
- Please, correct the location of the percentages in the last sentence of the “Physical activity level and incidence of low birthweight” section. It can be misunderstanded: “With regard to the level of sedentary physical activity, 34 (15.7% REMOVE) (ADD 94.4%) and 26 (16.4% REMOVE) (ADD 72.2%) of the mothers who gave birth to LBW babies were those who sat or reclined for <165 minutes per day (HERE 15.7%) and slept ≥8 hours (HERE 16.4%), respectively”.
- Please, do not refer to sleep as sedentary physical activity, since sleeping is not a type of physical activity.
- Please, correct the sentence in page 16, line 268: “The risk of LBW was 2.6 times higher in mothers who were in their third trimester (ADD: AND PERFORMED SQUATTING?) than those who did not perform squatting”.
